# Abnormal Chest X-Ray Findings of Patients With Confirmed Tuberculosis in Ghana

**DOI:** 10.7759/cureus.86359

**Published:** 2025-06-19

**Authors:** Augustina Badu-Peprah, Yaa D Appiah, Jimah Bashiru Babatunde, Zeleke Alebache, Stephanie J Badu-Peprah, Frank Quarshie

**Affiliations:** 1 Department of Radiology, Kwame Nkrumah University of Science and Technology, Kumasi, GHA; 2 Department of Radiology, Komfo Anokye Teaching Hospital, Kumasi, GHA; 3 Department of Radiology, Korle Bu Teaching Hospital, Accra, GHA; 4 Department of Radiology, School of Medical Sciences, University of Cape Coast, Cape Coast, GHA; 5 Department of Research, Monitoring, and Evaluation, National TB Control Programme, Accra, GHA; 6 Department of Surgery, Komfo Anokye Teaching Hospital, Kumasi, GHA; 7 Research Directorate, Klintaps University College of Health and Allied Sciences, Tema, GHA; 8 Department of Mathematical Epidemiology, African Institute for Mathematical Sciences, Accra, GHA

**Keywords:** chest x-ray (cx-ray), early identification and diagnosis, ghana, lung lesions, tuberculosis

## Abstract

Background

Tuberculosis (TB) remains a major global health issue despite being treatable and preventable. Early diagnosis and effective treatment are crucial for reducing TB transmission, in line with global control strategies. Chest X-rays, widely accessible and rapid, are key imaging tools for diagnosing TB. This retrospective study sought to assess chest X-ray findings of patients with confirmed tuberculosis in Ghana.

Methods

This study retrospectively analyzed chest X-ray findings to characterize lung lesions and assess tissue damage in 131 patients with bacteriologically confirmed TB. Lung lesions were categorized based on parenchymal changes, lesion extent, location, and pleural involvement.

Results

Reticulonodular infiltrates were observed in 66.4% (n=87) of patients, consolidation/ground-glass opacities in 58.8% (n=77), reticular infiltrates in 45.0% (n=59), and cavities in 42.0% (n=55). Pleural effusions were noted in 31.3% (n=41), predominantly unilateral (24.4%, n=32). Parenchymal anomalies primarily affected the apical/upper lung zones, mostly on the left (70.2%, n=92). Both middle-zone (48.1%, n=63) and lower-zone (49.6%, n=65) abnormalities predominantly affected the right lung. The extent of lung involvement varied: 23.6% (n=31) had small (one zone), 11.4% (n=15) medium (two zones), and 58.0% (n=76) large (more than two zones) involvement.

Conclusion

These findings highlight the significant lung damage caused by TB and underscore the importance of imaging in early detection and management.

## Introduction

Tuberculosis (TB) poses a significant global health challenge despite the efforts of various health organizations to combat it. The United Nations (UN) Sustainable Development Goals (SDGs) include a health target to end the TB epidemic by 2030 [1]. Achieving this goal requires a combination of early diagnosis and effective treatment to reduce TB transmission, morbidity, and mortality. TB is an infectious disease caused by the *Mycobacterium tuberculosis* bacterium, primarily transmitted through inhaling airborne droplets containing the bacteria. The most common form of TB is pulmonary tuberculosis (PTB), which typically serves as the initial site of infection. However, TB can also affect other parts of the body, including the spine, abdomen, and brain [1,2].

The progression of TB following inhalation of the bacteria depends largely on the individual's immune system. In about 5% of infected individuals, the immune system fails to control the initial infection, leading to the development of active TB within the first two years. This condition is known as primary TB [2,3]. Conversely, another 5% of infected individuals successfully suppress the initial infection, but viable mycobacteria persist in a latent state. These latent bacteria can reactivate later in life when the immune system becomes compromised, leading to post-primary TB. The remaining 90% of infected individuals fall into the category of latent TB infection, where they do not develop symptoms and remain non-contagious. The immune response to the mycobacteria significantly influences how TB manifests both clinically and radiologically [2,4].

Common symptoms of active TB include a persistent cough lasting more than two weeks (sometimes with hemoptysis), chest pain, weight loss, fever, night sweats, and fatigue [2,4,5]. Radiologically, primary TB is characterized by signs such as lymphadenopathy, consolidation, pleural effusion, and miliary nodules. Post-primary TB, on the other hand, is typically associated with nodules, cavitation, and consolidations, predominantly affecting the apical and upper lung zones [3].

Chest imaging plays a crucial role in the diagnosis and management of TB, with chest X-rays (CXRs) serving as the primary imaging modality due to their speed and accessibility compared to other imaging techniques like Computed tomography (CT) scans [3,6]. The sensitivity of CXRs is noteworthy, increasing from 85% in cases with suggestive TB abnormalities to 94% when any abnormality is detected, as highlighted in the World Health Organization’s (WHO) consolidated TB guidelines of 2021 [2,3,7]. This high sensitivity makes CXRs an excellent screening tool for TB. Beyond detecting lung abnormalities consistent with PTB, CXRs are used in several important ways during the diagnosis and management of TB, such as differentiating between active and latent TB; monitoring treatment outcomes; ruling out differential diagnoses, and many others [8,9].

In 2021, an estimated 10.6 million people developed TB, with about 1.6 million dying from the disease, including both adults and children, regardless of their HIV status [2,7]. Sputum culture and smear testing, although effective, require several days to yield results, delaying diagnosis and patient isolation, and thus contributing to continued TB transmission in the community [10,11]. These factors underscore the importance of CXR imaging in achieving global TB control targets, as outlined in the SDGs. However, the effectiveness of CXR as a diagnostic tool is contingent upon the accurate interpretation of the images by healthcare professionals [7,10,11].

In Ghana, a national TB survey conducted in 2013 revealed that CXRs detected approximately 75% of TB cases, with nearly 60% potentially being missed if only symptom screening had been used [11-13]. Early diagnosis and treatment are critical in reducing the spread of TB and improving patient outcomes. While CXR imaging provides significant benefits, the accuracy of PTB diagnosis largely depends on the expertise of the physician or radiologist interpreting the images [14,15]. Hence, there is a need to highlight the radiological findings of TB. This study, therefore, sought to examine the CXR findings associated with PTB and systematically categorized these findings based on parenchymal changes, lung involvement extent, lesion location, and pleural involvement. The following were the specific objectives: (i) Assessment of the pattern of parenchymal changes in confirmed TB patients, (ii) Evaluation of the distribution of parenchymal changes in confirmed TB patients, and (ii) Assessment of other CXR characteristics of PTB.

## Materials and methods

Study design and area

This was a cross-sectional study that retrospectively reviewed CXRs of patients with bacteriologically confirmed TB. The CXRs analysed were taken during a nationwide cross-sectional survey conducted in Ghana in 2013 in which the TB prevalence in Ghana was calculated to be 356 per 100,000 population among adults and children over 15 years [13].

Study population

In the 2013 prevalence survey, there were 61,726 participants in total, of which 59,718 (96.7%) underwent only frontal (posterio-anterior (PA)) CXR, with the rest exempted due to pregnancy [13]. Of the participants who had a CXR scan, 5,158 participants (9%) had abnormal CXRs, making them eligible for sputum examination. A total of 202 participants were bacteriologically confirmed as having PTB in the survey. These individuals either reported a cough lasting two weeks or more and/or had abnormal CXRs. Sputum tests confirmed the presence of *Mycobacterium *species in at least one test. The current study comprised all patients from the 2013 national survey who were confirmed to have PTB.

Eligibility criteria

This study included all CXR images of individuals who were confirmed to have PTB through laboratory testing, based on positive smear microscopy, culture, or GeneXpert results in the 2013 study by Bonsu et al. [13]. All cases with confirmed PTB and available CXR images were eligible for inclusion. Participants who were bacteriologically confirmed to have PTB but with normal CXRs were excluded from this study.

Sample size and technique

Out of the 202 participants bacteriologically confirmed with pulmonary TB, 165 underwent CXR scanning, while 37 did not due to pregnancy. Of the 165, 34 were excluded from the study because no parenchymal abnormalities were detected. Consequently, a total of 131 CXR images were consecutively reviewed for this study, resulting in a power of 0.8216.

Data collection procedure and x-ray images review

Data were collected using a Google Form-based extraction tool (Google LLC, Mountain View, California, United States) and exported to Excel (Microsoft Corporation, Redmond, Washington, United States). The data encompassed variables such as parenchymal changes, the extent of lung involvement, lesion location, and pleural involvement. Frontal CXRs were systematically assessed for pulmonary and nodal features characteristic of tuberculosis. The parenchymal abnormalities evaluated included: (i) Cavities: well-demarcated radiolucent areas with or without air-fluid levels; (ii) Consolidation: homogeneous opacities obscuring vascular markings; (iii) Fibrosis: linear or reticular opacities with volume loss and displacement of fissures or hila; (iv) Bronchiectasis: tram-track or ring-like opacities; (v) Ground-glass opacities: areas of hazy increased opacity with preserved bronchovascular margins. Lung fields were divided into upper, middle, and lower zones bilaterally. The upper zone comprised the area from the apex to the second anterior rib, the middle zone was between the second and fourth anterior ribs, and the lower zone was from the fourth rib to the diaphragm. The extent of lung involvement was visually estimated for each zone and categorized as: mild (<25%), moderate (25-50%), and severe (>50%). Lymph node involvement was assessed indirectly by identifying: increased hilar opacity with a convex outer border, and a positive hilum overlay sign.

Pleural abnormalities were also assessed on the frontal (PA) CXR only, and classified as: (i) Pleural effusion, seen as homogeneous opacity in the dependent portion of the hemithorax, with a concave upper border (meniscus sign) and obliteration of the costophrenic angle; (ii) Pleural thickening, a non-homogeneous linear or curvilinear opacity along the pleural surface, without meniscus or associated adjacent parenchymal fibrosis were excluded; Pleural effusion was graded based on extent of visualised pleural opacity on the frontal chest X-ray as follows: (i) Mild: pleural opacity limited to the costophrenic angle with blunting of the costophrenic angle only; (ii) Moderate: pleural opacity involving one-third to two-third of the hemithorax with opacity rising up the lateral chest wall but spares the upper third; and (iii) Severe: pleural opacity involving more than two-third of the hemithorax, massive opacity with mediastinal shift or near-complete hemithorax opacification.

The imaging studies were independently reviewed and reported by three radiologists (ABP, YDA, JBB). To minimize interobserver variability, a feature was considered valid if at least two radiologists agreed on it. In cases of disagreement, a fourth radiologist (ATA) was consulted, ensuring that the final data accurately represented the intended measurements and minimizing errors in reporting.

Data analysis

STATA 14.1 (released 2015; StataCorp LLC, College Station, Texas, United States) was used for statistical analysis of the data. The analysis focused on identifying CXR findings in patients with confirmed PTB, including the anatomical distribution of the lesions, the extent of pulmonary involvement based on the affected zones, and the categorization of TB based on radiographic patterns. Descriptive statistics, such as frequencies and percentages, were used to examine and describe the distribution of the study's major characteristics.

Ethics approval

The study was approved by the Komfo Anokye Teaching Hospital Institutional Review Board (approval number: KATHIRB/AP/198/23). Permission was received from Dr. Frank Adae Bonsu, the Principal Investigator of the 2013 TB Prevalence Survey, Former Programme Manager, National Tuberculosis Programme, before the data was used for this study. Patient data from the survey were anonymized and de-identified before analysis.

## Results

A total of 131 CXRs with parenchymal abnormalities from participants with confirmed PTB were reviewed. The mean age of the PTB patients with abnormal CXR findings was 53.47±7.89 years, and male patients constituted the majority (80.2%, n=105). PTB was more common in the age group of 55-64 years age group (28.2%, n=37) and those in the urban areas (65.6%, n=86) (Table 1).

**Table 1:** Demographic characteristics of pulmonary tuberculosis patients with abnormal chest X-ray findings

Variable	Frequency	Percentage
Sex		
Male	105	80.2%
Female	26	19.8%
Age Group (in years)		
15-24	8	6.1%
25-34	5	3.8%
35-44	15	11.5%
45-54	34	26.0%
55-64	37	28.2%
>64	32	24.4%
Mean age	53.47±7.89 years	
Residence		
Urban	86	65.6%
Rural	45	34.4%

**Figure 1:**
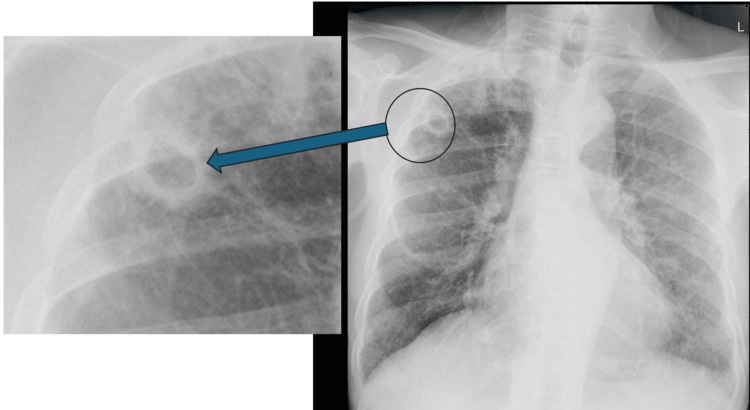
Postero-anterior chest x-ray image of a 65-year-old male patient with more than two weeks history of cough shows a right upper zone thick-walled cavity (blue arrow), indicative of active tuberculosis. Image Credit: Field data from the 2013 National TB prevalence survey. Permission was obtained from Dr. Frank Adae Bonsu, the Principal Investigator and the original data custodian of the 2013 National TB Prevalence Survey, to use the image.

The most common parenchymal abnormalities identified were reticulonodular infiltrates, consolidation/ground glass opacity, reticular infiltrates, and cavities (Figure 1). Reticulonodular infiltrates were present in 87 patients (66.4%), consolidation/ground glass opacities in 77 patients (58.8%), reticular infiltrates in 59 patients (45.0%), and cavities in 55 patients (42.0%).

**Figure 2:**
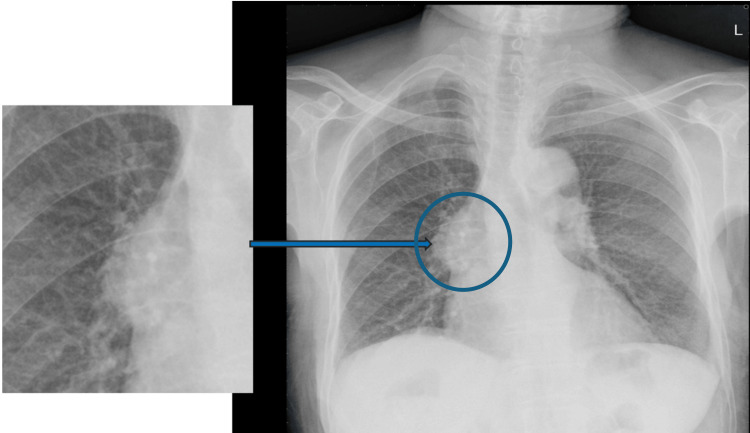
Postero-anterior chest x-ray of a 73-year-old male patient with no history of cough or previous TB shows a right perihilar mass with overlay sign (blue arrow) indicative of lymphadenopathy. Image Credit: Field data from the 2013 National TB Prevalence Survey. Permission was obtained from Dr. Frank Adae Bonsu, the Principal Investigator and the original data custodian of the 2013 National TB Prevalence Survey, to use the image.

Table 2 outlines the pattern of parenchymal changes observed in the study. Less common findings included miliary nodules (3.8%), perihilar lymphadenopathy (Figure 2) (3.1%), tuberculomas (1.5%, n=2), and traction bronchiectasis (7.6%, n=10). Notably, persistent calcification (Ghon’s focus) was absent in all patients.

**Table 2:** Pattern of parenchymal changes in confirmed tuberculosis patients Note: Yes= presence of parenchymal changes; No= absence of parenchymal changes

Parenchymal abnormality	No (N=131), n (%)	Yes (N=131), n (%)
Cavity	76 (58.0%)	55 (42.0%)
Consolidation/ ground glass opacity	51 (41.2%)	77 (58.8%)
Miliary nodules	126 (96.2%)	4 (3.8%)
Perihilar lymphadenopathy	127 (96.9%)	4 (3.1%)
Reticulonodular infiltrates	44 (33.6%)	87 (66.4%)
Reticular infiltrates	72 (55.0%)	59 (45.0%)
Tuberculoma	129 (98.5%)	2 (1.5%)
Persistent calcification (Ghon’s focus)	131 (100.0%)	0 (0.0%)
Traction bronchiectasis	121 (92.4%)	10 (7.6%)
Apical lung zone volume loss	117 (89.3%)	14 (10.7%)
Peribronchial fibrosis	125 (95.4%)	6 (4.6%)

The apical/upper lung zones were the most frequently affected by parenchymal abnormalities, with 92 patients (70.2%) showing involvement on the left side and 84 patients (64.1%) on the right. The middle and lower lung zones were also affected, though to a lesser degree. In the middle lung zone, abnormalities were observed in 60 patients (45.8%) on the left and 63 patients (48.1%) on the right. For the lower lung zone, 61 patients (46.6%) had abnormalities on the left, while 65 patients (49.6%) had them on the right. Table 3 provides a detailed breakdown of the distribution of these abnormalities across different lung zones.

**Table 3:** Distribution of parenchymal changes in confirmed tuberculosis patients Note: Yes= presence in location; No= absence in location

Location	Left (n=131)		Right (n=131)	
	No, n (%)	Yes, n (%)	No, n (%)	Yes, n (%)
Apical/Upper lung	39 (29.8%)	92 (70.2%)	47 (35.9%)	84 (64.1%)
Middle lung zone	71 (54.2%)	60 (45.8%)	68 (51.9%)	63 (48.1%)
Lower lung zone	70 (53.4%)	61 (46.6%)	66 (50.4%)	65 (49.6%)

Regarding the extent of lung involvement, Table 4 indicates that 31 patients (23.6%) had small involvement (affecting one zone), 15 patients (11.4%) had medium involvement (two zones), and 76 patients (58.0%) had large involvement (more than two zones). Pleural effusions were present in 41 patients (31.3%), with unilateral effusions (24.4%, n=32) being more common than bilateral effusions (6.9%, n=9). As for the extent of pleural disease, 36 patients (27.5%) had minimal effusion, five patients (3.8%) had moderate effusion, and none showed signs of massive pleural effusion (Table 4).

**Table 4:** Other chest x-ray characteristics of pulmonary tuberculosis Note: Yes= presence of characteristics; No= absence of characteristics

Variables	No (n=131), n (%)	Yes (n=131)
Extent of lung involved		
Small (One zone)	100 (76.3%)	31 (23.6%)
Medium (Two zones)	116 (88.6%)	15 (11.4%)
Large (More than 2 zones)	55 (42.0%)	76 (58.0%)
Pleural effusion present		
Unilateral	99 (75.6%)	32 (24.4%)
Bilateral	122 (93.1%)	9 (6.9%)
Extent of effusion		
Minimal	95 (72.5%)	36 (27.5%)
Moderate (Up to middle lung zone)	126 (96.2%)	5 (3.8%)
Massive	131 (100.0%)	0 (0.0%)

## Discussion

This study analyzed the CXR of 131 patients with confirmed PTB who presented with abnormal CXRs, focusing on the distribution and nature of parenchymal changes. It showed a higher prevalence of PTB in male participants, older age groups, and urban areas, as reported by Bonsu et al. [13]. The reason for the higher prevalence in urban areas could be due to overcrowding, as urban areas often experience overcrowding, which increases the risk of transmission. The most common abnormalities observed were reticulonodular infiltrates (66.4%), consolidation/ground-glass opacities (58.8%), reticular infiltrates (45.0%), and cavities (42.0%). These findings are consistent with the existing literature [8,9], where reticulonodular infiltrates and consolidation are frequently highlighted as key radiological features of PTB.

The study emphasizes the diverse range of parenchymal abnormalities in TB patients in Ghana [13]. The high prevalence of reticulonodular infiltrates and consolidation/ground-glass opacities is in line with the known pathophysiology of TB, where inflammation from the infection leads to these characteristic lung changes [8]. The presence of cavitary lesions in 42.0% of participants is significant, reflecting similar rates reported in studies from Nigeria [9], Thailand [16], and South Africa [17], where cavity prevalence ranges from 38% to 53.5%. Cavities are particularly significant as they often signal more advanced or severe disease, which is associated with poorer outcomes, drug resistance, and a higher risk of treatment relapse. Additionally, they increase the risk of transmission by facilitating the release of bacilli into the airways [18].

Less common abnormalities, such as miliary nodules and perihilar lymphadenopathy, were found in a small proportion of patients, demonstrating the broad spectrum of TB’s radiographic presentations [16]. Abnormalities were predominantly located in the apical/upper lung zones, with 70.2% of patients showing changes on the left and 64.1% on the right. This distribution is consistent with the classical understanding of post-primary TB, which typically affects the upper zones due to the higher oxygen concentration that favors *M. tuberculosis* growth [18]. In contrast, abnormalities in the middle and lower lung zones were less common, affecting fewer than 50% of patients, reflecting the disease's progression from the upper to lower lung regions.

The extent of lung involvement varied, with 58.0% of patients showing large areas of affected lung tissue, suggesting that many were diagnosed at an advanced disease stage [9]. This highlights the importance of early detection and intervention. Pleural effusions were present in 31.3% of patients, with unilateral effusions (24.4%) being more common than bilateral (6.9%). Most pleural effusions were minimal, consistent with previous studies, which suggest that severe effusions are less common in TB patients [6,16].

Limitations

The sample size may be limited, which could affect the generalizability of the findings to the larger population. The study's findings may not be generalizable to other settings or populations, such as patients with different demographic characteristics or comorbidities. These limitations should be considered when interpreting the study's findings and applying them to clinical practice or policy development.

## Conclusions

Overall, this study addresses a gap in the literature by providing a detailed characterization of CXR findings in confirmed TB cases in Ghana. It serves as a valuable resource for clinicians and public health professionals, enhancing the understanding of TB's radiographic manifestations in this setting. Future research should aim to correlate these radiographic findings with clinical outcomes to refine diagnostic criteria and optimize treatment strategies. These findings will help clinicians better understand the radiological spectrum of tuberculosis and tailor patient care more effectively. The fact that some participants with bacteriologically confirmed PTB had normal CXRs further emphasizes the need for laboratory testing in patients with strong clinical suspicion of TB, even when CXR results are normal.

The study highlights the critical need for early detection, as many patients presented with advanced disease, marked by extensive lung involvement and pleural effusions. This underscores the importance of enhancing TB screening and diagnostic practices to enable earlier detection and treatment, which would help reduce transmission rates and improve patient outcomes.
